# Knowledge of Childhood Autism and Challenges of Management among Medical Doctors in Kaduna State, Northwest Nigeria

**DOI:** 10.1155/2015/892301

**Published:** 2015-03-23

**Authors:** E. E. Eseigbe, F. T. Nuhu, T. L. Sheikh, P. Eseigbe, K. A. Sanni, V. O. Olisah

**Affiliations:** ^1^Department of Paediatrics, ABUTH, Shika-Zaria 810001, Nigeria; ^2^Federal Neuro-Psychiatric Hospital, Kaduna, Nigeria; ^3^Department of Family Medicine, ABUTH, Shika-Zaria 810001, Nigeria; ^4^Department of Psychiatry, ABUTH, Shika-Zaria 810001, Nigeria

## Abstract

Autism is a neurodevelopmental disorder with serious implications in childhood. There is a significant gap in the identification and provision of health and social services for autism in Africa. The knowledge of autism among health care providers and identifying challenges associated with its management could facilitate bridging the gap and ensuring better outcomes. A self-administered tool, the Knowledge about Childhood Autism among Health Workers (KCAHW) questionnaire, was used in assessing knowledge of autism among 175 medical doctors (participants) attending an annual scientific meeting in northwest Nigeria. Other parameters assessed were sociodemographic and professional characteristics of the participants and challenges encountered in the management of autism. Out of 175 questionnaires distributed, 167 (95.4%) were returned. Good knowledge (KCAHW score ≥15) was significantly associated with being a paediatrician or psychiatrist and practicing in a tertiary health facility (*P* < 0.05), while poor knowledge (KCAHW score <15) was significant among general practitioners (*P* < 0.05). The highest knowledge gap was associated with onset of autism and its comorbidities (KCAHW Domain 4) while the least was concerning communication impairments (KCAHW Domain 2). Major challenges encountered in autism management were dearth of specialist services, cost of evaluation, and poor caregiver perspectives of autism.

## 1. Introduction

Autism spectrum disorder (ASD) is a spectrum of neurodevelopmental disorders occurring early in childhood that is characterized by persistent deficits in social communication and interaction and restricted, repetitive patterns of behavior, interests, or activities [1]. In the latest Diagnostic and Statistical Manual of Mental Disorders (DSM 5), autism is one of the disorders classified as ASD, the others being Asperger syndrome and pervasive developmental disorder not otherwise specified [1]. The global median prevalence of autism is estimated at 17/10,000 [2]. In Africa, reports on autism are mainly from neurology and psychiatric clinics and indicate a prevalence ranging from 0.7% to 33.6% among cases seen in these clinics [3]. Early recognition and intervention as well as multidisciplinary specialist care services, which facilitate positive outcomes, characterize the management of autism in developed climes [4]. However, dearth of relevant epidemiological studies and data, limited knowledge among health care providers, poor community awareness, and a dearth of specialist care services are some of the issues confronting autism in Africa [3, 5].

Assessment of the knowledge of autism and challenges encountered in its management among health care providers would help in addressing issues confronting the disorder. In resource limited settings like ours, this would promote awareness, facilitate early diagnosis and intervention, and improve quality of care and outcomes in autism.

Studies on autism from Nigeria indicate a low level of autism knowledge and awareness even among health care workers [3, 6–8]. The majority of these studies have been from south eastern Nigeria and among health workers other than medical doctors. Medical doctors play a pivotal role in health care delivery in Nigeria. Their knowledge of autism, in any health care setting, is crucial to the provision of optimal services and reduction of the burden associated with autism. This is the first study, to the best of our knowledge, which assesses the knowledge of autism and management challenges among medical doctors in northern Nigeria. The aim of study was to highlight any knowledge gap and management challenges encountered, with a view to addressing them and consequently improving autism outcomes in our environment.

## 2. Methods

The participants in this study were medical doctors who attended the 2013 Annual General Meeting (AGM) of the Kaduna State Chapter of the Nigerian Medical Association (NMA). Kaduna State, one of the 36 states and the federal capital territory that make up Nigeria, is located in Northwest Nigeria and has a population of approximately 6.1 million persons. There are approximately 1000 registered doctors, a quarter of which are medical specialists, practising in the state. However, a majority of the specialists and over seventy percent of the doctors practice in the five tertiary health institutions that are located in the two major towns of the state. The Kaduna State NMA Chapter is one of the 37 chapters that make up the NMA. It holds an annual general meeting of its members. The study was conducted at the plenary session of the 2013 AGM. A total of 175 doctors were present at the session and all consented to the study.

The Knowledge about Childhood Autism among Health Workers (KCAHW) Instrument was used in assessing knowledge of autism among the participants. The KCAHW instrument is a basic knowledge of autism assessment tool designed by Bakare et al. in 2008 [9]. It is a self-administered questionnaire that assesses autism knowledge by scoring nineteen items in four domains, namely, social interaction (Domain 1), impairment in communication (Domain 2), obsessive and repetitive behaviour (Domain 3), characteristics of autism as a disorder and its comorbidities (Domain 4) [9]. The cumulative KCAHW score is 19 (Table 1).

For the purpose of this study the median KCAHW score of the participants was identified as the benchmark of knowledge among the participants. The median score provides a valid measure of central tendency. Consequently those who scored less than the median score were classified as having poor knowledge among the participants while those with a score equal to or greater than the median were classified as having good knowledge. Also knowledge gap was identified if any participant's total score in a domain was suboptimal.

Other parameters of the participants assessed included age, sex, years of practice, place of practice, area of specialization, history of autism case management, and challenges encountered in autism management.

Informed consent was obtained from each participant and the study was approved by the research ethics committee of the Federal Neuro-Psychiatric Hospital (FNPH), Kaduna.

Data was analysed for mean and median distribution of KCAHW scores. Chi square test was used in determining the relationship between participants' characteristics and their KCAHW scores. A *P* value of <0.05 was regarded as significant.

## 3. Results

A total of 175 questionnaires were distributed and 167 (95.4%) were returned. There was a male preponderance (119, 71.3%) among the participants whose age ranged from 26 to 60 years (mean 37.5 ± 8.9 years). The years of practice ranged from 1 to 33 years (mean 10.2 ± 8.8 years, median 8 years). Most (91, 54.5%) of the participants practiced in a tertiary health facility and the same number of participants were either specialists or specialists in training. Out of the 76 (44.5%) participants who were general practitioners, 31 (40.8%) were females.  Table 2 shows the distribution of the participants according to their area of specialty.

The mean and median KCAHW scores of the participants were 13.5 ± 3.7 and 15, respectively. The KCAHW Domain 2 which assesses impairments in communication had the highest number (123, 73.7%) of participants with maximum score while Domain 4 which assesses characteristics of autism as a disorder and its comorbidities had the least (18, 10.8%). Maximum scores in Domains 1 (impairments in social interactions) and 3 (obsessive and repetitive pattern of behaviour) were obtained by 46 (27.5%) and 58 (34.7%) participants, respectively. The main item in each of the domains where most participants demonstrated knowledge gap (wrong or do not know response warranting a score of 0) is shown in Table 3. Good knowledge of autism (KCAHW score ≥15) among the participants was significantly (*P* < 0.05) associated with male sex, having seen a case of autism, specialist practice particularly the medical subspeciality, and practice in a tertiary health facility (Table 4).

Fifty-eight (34.7%) participants had seen a case of autism out of whom 20 (34.5%) were general practitioners and 38 (65.5%) were specialists. Mode of diagnosis was by clinical suspicion in 39 (67.2%) of the participants and the use of Diagnostic and Statistical Manual- (DSM-) IV classification by the others (19, 32.8%). The same participants who used the DSM-IV in diagnosis managed their cases while the other 39 (67.2%) participants referred their case to either a paediatrician (22, 56.4%) or psychiatrist (17, 43.6%). Modalities for management employed by the participants were counselling of parents (19, 100%), occupational therapy (4, 21.1%), treatment of associated seizures (2, 10.5%), and referral to a special education facility (15, 79%). Lack of speech and behavioural therapists was a major challenge in management of autism (Table 5).

## 4. Discussion

Participants displayed an appreciable knowledge of autism and this was significantly associated with specialist practice and attending to a case of autism in the past. Dearth of specialist intervention services and cost of management as well as poor caregiver perspectives were the major challenges of autism management identified.

There is a paucity of epidemiological data on autism from Africa [3, 5]. Studies from Nigeria indicate a poor level of awareness and knowledge of the disorder even among health care workers, lack of therapeutic services such as speech and behavioral therapists, and a negative attitudinal disposition towards persons with autism [3, 6–13]. Most of these studies were conducted in clinical and educational settings which limits generalization of their outcomes. Despite their limitations, most of these studies demonstrate a high prevalence of nonverbal autism cases, occurrence of multiple and severe comorbidities with autism, and a significant delay before diagnosis or presentation at health care facilities [11–13]. Studies indicative of a poor knowledge of autism among health care providers have also been reported from other parts of the world. Rahbar et al. in their study among a population of general practitioners in Pakistan noted that 44.6% of the doctors had only heard of autism [14]. Even where health care providers display a high level of awareness they still exhibited misconceptions about autism [15]. Nonetheless a better knowledge of autism has been associated with specialization in psychiatry and paediatrics, place of work, and age of practice [6–9].

Improving knowledge and awareness on autism has been identified as vital to addressing issues confronting autism in Africa [3, 5]. Assessment of the autism knowledge gap, particularly among care providers in autism, is paramount to bridging this gap. The KCAHW, whose use has been validated, has been found to be useful in identifying basic deficiencies in the knowledge of autism among health workers [9]. A variable knowledge of autism, following assessment with the KCAHW tool, has been reported severally among health care providers in Nigeria [6, 8]. Bakare et al. [6] and Igwe et al. [8] reported mean KCAHW scores lower than scores in this study and indicative of a lesser knowledge of autism, among health care providers in eastern Nigeria. Higher mean KCAHW scores in this study could be a reflection of the fact that this study was conducted among medical doctors as against nurses and other health workers in other studies. The depth of medical training and practice is likely to predispose the medical doctor to knowing more about autism than other health workers, in the same health system.

The widest knowledge gap was regarding comorbidities such as seizures, associated with autism, and the period of onset of the disorder in childhood. Almost a third of the participants (31.1%) were either wrong or unsure about seizures in autism and the period of onset in autism. The presence of a gap in the knowledge of comorbidities associated with autism as well as with regard to the onset of autism has been reported among health care providers in the United States and Pakistan, respectively [15, 16]. Hartley-McAndrew et al. in their study on knowledge of autism spectrum disorders in potential first-contact professionals reported that 46% of the professionals were unsure as to whether seizures were commoner (which is a fact) in children on the autism spectrum [15]. Also, Imran et al. reported that 43.6% of physicians in their study did not feel that onset before 36 months (a diagnostic hallmark of autism) was necessary for a diagnosis of autism [16].

Knowledge gaps in KCAHW domains in this study and other related studies could suggest a deficient autism education in the professional formative years of the health care providers. In a survey conducted among paediatricians in Nigeria concerning the teaching of autism in medical schools, sixteen medical schools were randomly selected from the twenty-nine registered schools and it was observed that none offered autism as a specific lecture topic (R. D. Wammanda and E. E. Eseigbe, personal communication, 3 April 2014). This could significantly limit the knowledge of autism among medical graduates from these schools. This finding and its implication were highlighted at the First Autism in Africa Conference that took place in Ghana and was organised by the International Child Neurology Association (ICNA) to tackle autism in the continent, in April 2014.

The pattern of knowledge distribution among the participants is consistent with previous findings that indicate a better knowledge of autism among those in the specialties and practice of paediatrics and psychiatry [6, 8]. Behavioral abnormalities, intellectual and neurodevelopmental delays, and feeding difficulties are some complaints associated with autism [16]. These complaints often necessitate visits to the psychiatrist or paediatrician. It becomes professionally expedient for this group of specialists to be knowledgeable about diseases, including autism, which could present with these complaints. This could explain the significant knowledge of autism among participants that were in these specialties. This could also explain why those who were in the surgical subspeciality and who are less likely to see cases of autism were significantly less knowledgeable than their medical counterparts. However, it is important to note that evaluation in autism management and impairments in communication as well as social interactions could lead to the consultation of the neurosurgeon or otolaryngologist. Consequently limited knowledge of autism among surgical specialists as observed in this study has serious implications for diagnosis and intervention in autism management. It would also represent underutilization of scarce specialist resources.

Having a better knowledge of autism was also associated with working in a tertiary health institution. Cases of autism are more likely to be referred to such institutions for expert management. This could have influenced the significant knowledge associated with working in such institutions. It could also explain why general practitioners, working in lower levels of health care delivery, were significantly less knowledgeable about autism. Furthermore, the fact that the majority of the female participants were general practitioners could have accounted for the difference in autism knowledge among the sexes.

The challenges identified by the participants in the management of autism are typical of challenges encountered in the care of children with special health care needs in Africa [17]. Availability of health services and facilities for neurodevelopmental disorders in Africa is limited [3, 5, 6]. Furthermore, poor awareness, strong traditional beliefs, weak health systems, prevailing poor socioeconomic status, and lack of any institutionalized social support system tend to promote and sustain the enumerated challenges in the continent [3, 17, 18]. Specifically, issues that have been identified as confronting management of autism in Nigeria are similar to the challenges indicated by the participants and include negative attitudinal disposition towards autism such as the rejection of children with autism even in special education schools, lack of educational services and facilities for the affected children, lack of early diagnostic and interventional services for autism in our health care delivery system, and family disharmony as a result of the impact of the burden of autism and the lack of institutionalized social support [11–13, 18]. The high cost of patient evaluation was another major challenge indicated. The need to rule out other possible conditions that share similar symptoms with autism and determine the presence and severity of comorbidities could magnify the cost of evaluation. Other contributory factors could include lack of inclusion in a supportive health insurance scheme and the burden of other ancillary costs such as those incurred during repeated transport to and accommodation at specialist health centres where the facilities for evaluation are available. The economic challenge constituted by the management of autism is not limited to Nigeria or developing countries alone. It has been estimated that the lifetime cost for an individual with autism is estimated to be between $1.7 million [19] and $4.0 million [20] in developed countries necessitating the need for institutionalized support. High default rate among patients with special needs as encountered in autism and in this study has been reported severally from Nigeria [21]. Poor knowledge of the disorders among caregivers, high cost of evaluation, lack of specialist services, caregiver's dissatisfaction with outcome of management, and poor outcomes associated with the disorders are some factors that have been identified as contributory [21, 22].

The implications of gaps in autism knowledge among health care providers and the burdensome management challenges include misdiagnosis, delayed diagnosis and intervention, caregiver use of multiple and sometimes detrimental health care options, increased cost of care whilst shopping for remedies, increased family burden and disharmony, and ultimately poorer autism outcomes [3, 11–13, 18]. Bridging the knowledge gap and attenuating management challenges have the potential of improving autism outcomes even in resource limited settings like ours. Achieving these lofty objectives would require a multifaceted approach which should include ensuring basic autism education in medical curricula and training of first-point health care providers in the diagnosis of autism [8, 15]. Training of nonspecialists to provide therapeutic interventions in community settings as identified by Reichow et al. [23] has been advocated in resource limited settings in order to obviate challenges in the management of autism [24]. Additionally, there should be formulation and implementation of governmental health policies that promote autism awareness, identify autistics, and provide comprehensive care and support to persons with autism and their families. Furthermore, national initiatives should be integrated with regional and global autism awareness and management programmes. At the international level initiatives that tackle autism issues particularly in resource limited settings like ours, such as the ICNA sponsored First Autism in Africa Conference, should be encouraged.

## 5. Limitations

The study was limited to medical doctors who attended the NMA Meeting. Those who did not attend the meeting could have been more or less knowledgeable about autism. However, the characteristics of the attending doctors provide an insight into how doctors in the environment would respond to queries on autism.

## 6. Conclusion

The study showed a good knowledge of autism among medical doctors who are specialists particularly paediatricians and psychiatrists and in those who had seen a case of autism in the past. Knowledge was limited in general practitioners and the knowledge gap was mostly about onset and comorbidities of autism. Dearth of specialist services, cost of accessing care, and poor caregiver perspectives were major challenges of management. The study highlights the need to improve the knowledge of childhood autism among medical doctors and address the challenges hampering its management. These would increase the level of autism awareness and facilitate the achievement of better outcomes in the region.

## Figures and Tables

**Table 1:** KCAHW questionnaire's domains, items, and scores [4].

KCAHW domain	Knowledge assessed	Number of items	Total score
1	Impairments in social interactions	8	8
2	Impairments in communication	1	1
3	Obsessive and repetitive pattern of behaviour	4	4
4	Type of disorder autism and associated comorbidity	6	6

KCAHW = Knowledge about Childhood Autism among Health Workers.

**Table 2:** Participants, their specialties, and KCAHW scores.

Specialty	Number of participants *N* = 167 (%)	KCAHW scores	
		≥15 (*N* = 85^*^)	<15 (*N* = 82^*^)
General practitioner	76 (46)	26 (34)	50 (66)
Paediatrics	16 (10)	16 (100)	0
Psychiatry	13 (8)	13 (100)	0
Ophthalmology	12 (7)	5 (42)	7 (58)
OB-GYN	9 (5)	3 (33)	6 (67)
Public health	8 (5)	4 (50)	4 (50)
Internal medicine	8 (5)	5 (63)	3 (37)
Surgery	6 (4)	2 (33)	4 (67)
Otolaryngology	5 (3)	2 (40)	3 (60)
Radiology	4 (2)	2 (50)	2 (50)
Family medicine	4 (2)	3 (75)	1 (25)
Lab medicine	4 (2)	3 (75)	1 (25)
Anaesthesia	2 (1)	1 (50)	1 (50)

^*^Percent of Specialty.

**Table 3:** Items associated with major knowledge gaps in the KCAHW domains.

KCAHW domain	Main item	Number of participants scoring zero (%)
1	Absence of social smile in a child with autism	39 (23.4)

2	Delay or total lack of development of spoken language in autism	44 (26.3)

3	Association of autism with abnormal eating habit	48 (28.7)

4	Association of autism with comorbidities (mental retardation and/or epilepsy)	52 (31.1)

4	Onset of autism	52 (31.1)

**Table 4:** Relationship between participants and KCAHW scores.

Variable	Number of participants *N* = 167 (%)	KCAHW scores		*P* value
		≥15 (*N* = 85)	<15 (*N* = 82)	
Age (years)				
≥35	93 (55.7)	46 (54.1)	47 (57.3)	0.800
<35	74 (44.3)	39 (45.9)	35 (42.7)	
Sex				
Male	119 (71.3)	54 (63.5)	65 (79.3)	0.040
Female	48 (28.7)	31 (39.5)	17 (20.7)	
Years of practice				
≥8	91 (54.5)	49 (57.7)	42 (51.2)	0.500
<8	76 (45.5)	36 (42.3)	40 (48.8)	
Seen a case				
Yes	58 (34.7)	37 (43.5)	21 (25.6)	0.023
No	109 (65.3)	48 (56.5)	61 (74.4)	
Specialization				
Yes	91 (54.5)	59 (69.4)	32 (39)	0.000
No	76 (45.5)	26 (30.6)	50 (61)	
Place of practice				
Tertiary facility	91 (54.5)	63 (74.1)	28 (34.1)	0.000
Others	76 (45.5)	22 (25.9)	54 (65.9)	
Subspeciality	*N* = 91 (%)	≥15 (*N* = 53)	<15 (*N* = 38)	
Medical	59 (64.8)	44 (83)	15 (39.5)	0.000
Surgical	32 (35.2)	9 (17)	23 (60.5)	

**Table 5:** Major challenges in the management of autism as indicated by 19 participants.

Major challenge	Number of indicating participants (%)
Dearth of speech and behavioural therapists	19 (100)
High cost of patient evaluation	19 (100)
Poor understanding and acceptance of autism by caregivers	15 (79)
High default rate from follow-up care	10 (52.6)
